# Prevalence of rheumatic heart disease among school children in East Africa: a systematic review and meta-analysis

**DOI:** 10.11604/pamj.2021.38.242.26058

**Published:** 2021-03-08

**Authors:** Melaku Bimerew, Biruk Beletew, Addisu Getie, Adam Wondmieneh, Getnet Gedefaw, Asmamaw Demis

**Affiliations:** 1 Department of Nursing, College of Health Sciences, Woldia University, Woldia, Ethiopia,; 2 Department of Midwifery, College of Health Sciences, Woldia University, Woldia, Ethiopia

**Keywords:** Prevalence, rheumatic heart disease, school children, East Africa

## Abstract

**Introduction:**

conducting researches and estimating the prevalence of rheumatic heart disease in school children is crucial to develop school-based strategies targeted to decrease the prevalence of this disease. Therefore, this systematic review and meta-analysis were aimed to estimate the overall prevalence of rheumatic heart disease (RHD) among school children in East Africa.

**Methods:**

PubMed/MEDLINE, Google Scholar, Cochrane review, African Journals Online and African Index Medicus databases were searched to identify relevant research articles. The overall prevalence of rheumatic heart disease was pooled based on the weighted inverse variance random-effects model at a 95% confidence interval. The presence of heterogeneity, sensitivity analysis and presence of publication bias was tested. Results were presented with narrative synthesis, tables and forest plots.

**Results:**

a total of thirteen research articles were included in the final analysis. The pooled prevalence of rheumatic heart disease in East African school children was 1.79% (17.9 cases per 1000 children (95% CI=11.6, 24.2; I^2^=95.1%; p<0.001)). From the subgroup analysis conducted by publication year, a lower prevalence of RHD in school children was reported among studies published after 2015 (six studies; overall prevalence=1.17% (11.7 cases per 1000 school children); with 95% CI=0.60, 1.73%; I^2^=88.8%; p<0.001). Additionally, the horn of Africa was found to have the lowest prevalence of RHD in school children among East African countries (six studies; overall prevalence=1.59% (15.9 cases per 1000 school children); with 95% CI=0.68, 2.51%; I^2^=94.2%; p<0.001).

**Conclusion:**

the prevalence of rheumatic heart disease (RHD) among school children in East Africa was considerably higher than the results from high-income countries. Therefore, community education on strep throat and its complications should be implemented through mass media. Rheumatic heart disease preventive strategies should be integrated with schools to reduce the prevalence of RHD among school children.

## Introduction

Rheumatic heart disease (RHD) describes a group of acute or chronic cardiovascular conditions that are caused by rheumatic fever-an inflammatory disease triggered by group-A streptococcal infection. After 1-3 weeks, untreated or under-treated strep throat (streptococcal tonsillopharyngitis) results in humoral and cell-mediated inflammation of connective tissues including the heart, joints, brain and skin [1-4]. While other organs are mildly or transiently affected, inflammation of the heart may lead to death or life-long disabilities secondary to RHD which can take the form of valvular heart diseases, pericarditis, endocarditis, or heart block [5-7]. Valvular heart disease is the commonest form of RHD characterized by scarring and permanent damage of the heart valves; leading to valvular stenosis and regurgitation. The compensatory mechanisms attempted by the heart to overcome stenosis and regurgitation leads to congestive heart failure and finally death. Hence, RHD is a serious public health problem [4,5,8].

Worldwide, more than 30 million people are thought to be victims of RHD. It is also a cause of an estimated 305,000 deaths and 11.5 million disabilities. Besides, RHD is a significant cause of economic wastages, with an estimated annual expenditure of 5400 billion American dollars [9,10]. Rheumatic heart disease is an easily preventable condition. Three levels of prevention: the primordial, primary and secondary preventions, are known to be effective in decreasing its magnitude [2,5,11,12]. But, the predisposing condition to RHD (strep throat) is considered as a mild, self-limiting illness by most of the communities, especially in developing countries. Health seeking behavior and adherence to primary or secondary prevention of RHD remain low in those countries [13-16]. As a result, about 84% of RHD cases and 80% of estimated deaths are contributed from Africa, South-East Asia and Western Pacific regions [9].

Rheumatic heart disease can occur at any age, but school children (children aged from 5-15 years) are the highly affected groups. It causes school absenteeism, drop-out and premature deaths in those children [9,17]. Therefore, conducting researches and estimating the prevalence of RHD in school children is crucial to develop school-based strategies targeted to decrease the burden of this disease. Furthermore, researches from highly endemic areas (developing world) like East Africa are more important to reduce the global burden of the disease.

So far, many studies were conducted to assess the magnitude of RHD among school children in East African countries. But, reported magnitudes of RHD in those countries were inconsistent; ranging from 3 to 41 cases per 1000 school children [18,19]. Having a pooled result will help to overcome those inconsistencies and to have a common understanding. Despite this, no previous researches had estimated the overall magnitude of RHD among school children in East Africa. Hence, this meta-analysis was aimed to estimate the overall prevalence of RHD among school children in East Africa.

## Methods

**Reporting:** the Preferred Reporting Items for Systematic Review and Mata-Analysis (PRISMA) guideline was used to report this study [20].

**Searching strategies and information sources:** PubMed/MEDLINE, Google Scholar, Cochrane review, African Journals Online and African Index Medicus databases were searched to identify relevant research articles. Searching for grey literature from repositories and snowball searching were also employed to accommodate potentially related literature. The comprehensive searching strategy was developed according to Population Intervention Comparison and Outcome (PICO) standard questions (Table 1).

**Table 1:** the search strategies and information sources used to identify relevant research articles for this meta-analysis, 2020

Database	Search strategy/terms	Number of retrieved articles
PubMed/MEDLINE	Prevalence [title/abstract] or incidence [title/abstract] or burden [title/abstract] or magnitude [title/abstract] and rheumatic heart disease [title] or RHD [title]; then limiting the publication period from 1990 to 2020	1174
Google Scholar	Prevalence [title] or incidence [title] or burden [title] or magnitude [title] and rheumatic heart disease [title] or RHD [title] and infant or children or child or adolescent or schoolchildren or school children or pupil; then limiting the publication period from January 1990 to December 12/2020	220
Cochrane Review	Rheumatic heart disease in record title and prevalence in title abstract keyword or incidence in title abstract keyword or burden in title abstract keyword or magnitude in title abstract keyword; then limiting the publication period from January 1990 to December 12/2020	1942
African Journals Online	Prevalence or incidence or burden or magnitude and rheumatic heart disease [title/abstract] or RHD [title/abstract]	21
African Index Medicus	Prevalence [title/abstract] or incidence [title/abstract] or burden [title/abstract] or magnitude [title/abstract] and rheumatic heart disease [title] or RHD [title]	79
Others	Rheumatic heart disease and other related terms; by snowball methods etc.	16
Total articles retrieved		3452

**Study selection and eligibility criteria:** after retrieving, articles were exported to the endnote reference manager software version 7.0. to remove duplications. Two investigators (MB and AD) independently screened the selected articles by their titles and abstracts before retrieval of full texts. Published and unpublished cross-sectional studies that had reported the magnitude of RHD among school children in East African countries and published in the English language from 1990 to 2020 were included. But, citations, research articles with no accessible full text, commentaries, editorials and anonymous reports were excluded. After all, articles that met the inclusion criteria were reviewed in detail for their quality and their consistency with the objectives of this study. Findings from the included articles were summarized and reported using narrative synthesis and quantitative meta-analysis.

**Outcome variable:** prevalence of RHD in school children, which was pooled from research articles that had used: 1) either the World Health Organization (WHO) or World Heart Federation (WHF) criteria for defining RHD; and 2) both auscultation and echocardiography to diagnose RHD, was the outcome variable of this study. Additionally, the outcome variable of this study was pooled from research articles that had reported the total RHD cases or as a sum of definite and/or borderline or probable or possible cases of RHD; and in this meta-analysis, the final pooled result was reported as a total RHD case.

**Quality assessment:** after removing duplicate studies and screening potentially relevant articles, two independent authors (MB and BB) had appraised the quality of eligible articles by using the Newcastle-Ottawa Scale (NOS) for cross-sectional studies as a quality appraisal tool [21]. Disagreements between appraisers were solved by taking their mean scores. Studies scored 7 and above from the 10 item NOS were considered as low risk or high quality and included in the final analysis.

**Data extraction and statistical analysis:** the data was extracted and cleaned by using a Microsoft Excel worksheet; then was exported to STATA version 11.0 statistical software for further analysis. Standard errors for the prevalence of RHD were calculated using the binomial distribution formula. Then, the overall prevalence of RHD was pooled based on the weighted inverse variance random-effects model at a 95% confidence interval (CI). Results were presented by narrative synthesis and forest plots. Heterogeneity between included studies was assessed by Cochrane´s Q statistics (Chi-square), and inverse variance (I^2^) with p-values. Publication bias was assessed by funnel plots and Egger´s regression test. Sensitivity analysis was also conducted to observe if there is an influential study to affect the true value of the pooled prevalence of RHD.

## Results

**Figure 1:**
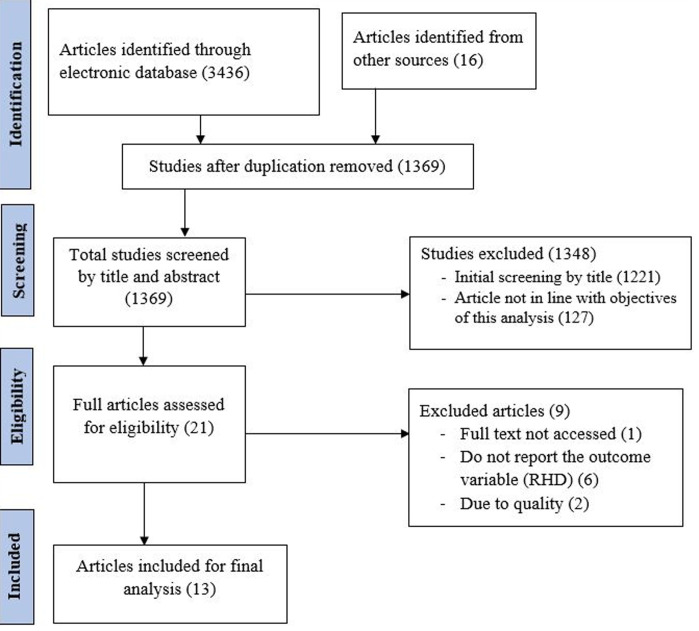
PRISMA flow diagram showing searching strategies, screening, reasons for exclusion and number of included research articles in this systematic review and meta-analysis, 2020

**Study selection and characteristics of the included studies:** a total of 3452 studies were retrieved from the electronic database searching. After screening and eligibility assessment (Figure 1), thirteen articles [18,19,22-32] were included for the final analysis. All of the included articles were cross-sectional in design. The age range of school children in each study was not similar but generally, it ranges from 4-24 years. All the included studies had used echocardiography to diagnose RHD after the initial screening with clinical examination (auscultation for heart murmur) (Table 2).

**Table 2:** characteristics of research articles included in this systematic review and meta-analysis, 2020

Authors' name (publication year)	Study area	Study area category	Quality score (10)	No of RHD (sample size)	Prevalence (cases/ 1000 schoolchildren)
Mulatu HA *et al*. (2016)	Ethiopia	Horn of Africa	9	6 (1847)	0.32 (3.25)
Engel ME *et al*. (2015)	Ethiopia	Horn of Africa	8	61 (2000)	3.05 (30.50)
Yadeta D *et al*. (2016)	Ethiopia	Horn of Africa	9	59 (3238)	1.82 (18.22)
Beaton A *et al*. (2012)	Uganda	Southeast Africa	9	72 (4869)	1.48 (14.79)
Mucumbitsi J *et al*. (2017)	Rwanda	Southeast Africa	8	17 (2501)	0.68 (6.79)
Rossi E *et al*. (2014)	Eritrea	Horn of Africa	7	28 (684)	4.09 (40.94)
Marijon E *et al*. (2007)	Mozambique	Southeast Africa	9	66 (2170)	3.04 (30.41)
Ali S *et al*. (2018)	Sudan	Western East Africa	8	29 (1515)	1.91 (19.14)
Musuku J *et al*. (2018)	Zambia	Southeast Africa	9	13 (1102)	1.18 (11.80)
Campanale CM *et al*. (2017)	Madagascar	Southeast Africa	9	7 (522)	1.34 (13.41)
Beaton A *et al*. (2015)	Uganda	Southeast Africa	9	192 (4773)	4.02 (40.20)
Oli K and Porteous J (1999)	Ethiopia	Horn of Africa	7	60 (9388)	0.64 (6.39)
Oli K *et al*. (1992)	Ethiopia	Horn of Africa	7	15 (3235)	0.46 (4.64)

RHD: rheumatic heart disease

**Figure 2:**
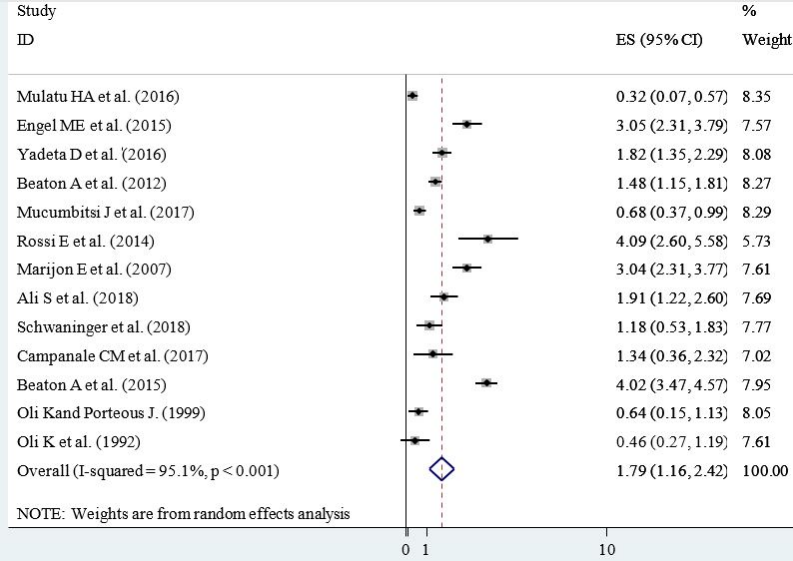
forest plot showing the prevalence of rheumatic heart disease per 100 school children in East Africa, 2020

**Prevalence of RHD in school children:** among the included studies, the lowest prevalence of RHD in school children was 0.32% (3.2 cases per 1000 children); while the highest was 4.09% (40.9 cases per 1000 children). The pooled prevalence of RHD among school children in East Africa was found to be 1.79% (17.9 cases per 1000 children (95% CI=11.6, 24.2; I^2^=95.1%; p<0.001)) (Figure 2).

**Heterogeneity:** the inverse variance (I^2^) was 95.1% with a p-value of <0.001 (Figure 2); suggesting the presence of heterogeneity on the reported prevalence of RHD among the included studies.

**Sensitivity analysis:** a leave-one-out sensitivity analysis was conducted to examine if the pooled prevalence of RHD in school children was greatly impacted by the result of a single study. But, all the results of this sensitivity analysis were within the 95% CI limits of the pooled prevalence (1.16-2.24%); suggesting the absence of an influential study that potentially affected the observed pooled prevalence of RHD in those children (Table 3).

**Table 3:** a leave-one-out sensitivity analysis among included studies showing if the pooled burden of rheumatic heart disease in school children was greatly impacted by the result of a single study, 2020

Study omitted	Estimate (%)	95% CI
Mulatu HA *et al*. (2016)	1.92	1.28, 2.57
Engel ME *et al*. (2015)	1.68	1.05, 2.32
Yadeta D *et al*. (2016)	1.79	1.11, 2.47
Beaton A *et al*. (2012)	1.82	1.11, 2.54
Mucumbitsi J *et al*. (2017)	1.89	1.19, 2.60
Rossi E *et al*. (2014)	1.65	1.02, 2.28
Marijon E *et al*. (2007)	1.68	1.05, 2.32
Ali S *et al*. (2018)	1.78	1.12, 2.44
Musuku J *et al*. (2018)	1.84	1.17, 2.51
Campanale CM *et al*. (2017)	1.82	1.16, 2.48
Beaton A et al. (2015)	1.56	1.04, 2.08
Oli K and Porteous J. (1999)	1.89	1.22, 2.57
Oli K *et al*. (1992)	1.90	1.24, 2.56
Overall	1.79	1.16, 2.42

CI: confidence interval

**Figure 3:**
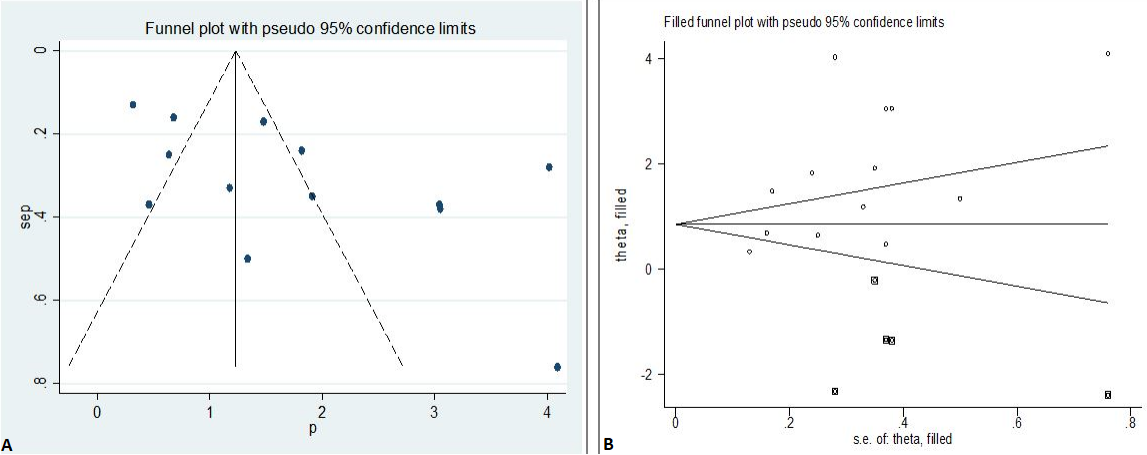
funnel plot showing publication bias (the asymmetrical distribution of included research articles by the prevalence of rheumatic heart disease) (A); and the employed trim-fill analysis to minimize publication bias (B), 2020

**Publication bias:** since the funnel plot had shown asymmetrical distribution (Figure 3A) and the Egger´s regression test was found to be significant with a p-value of 0.029, there was evidence of publication bias in the included studies. As a result, trim and fill analysis was conducted to trim the studies that cause asymmetry in the funnel plot and to fill imputed missing studies based on a bias-corrected overall estimate; so that the overall effect estimate produced by the remaining studies can be considered minimally affected by publication bias (Figure 3). Based on this trim and fill analysis (Figure 3B), five studies were filled and a total of eighteen studies were enrolled; resulting in a bias-corrected overall prevalence of RHD to be 0.91% (9.1 cases per 1000 school children (95% CI=0.22-1.60%)) using the random-effects model.

**Figure 4:**
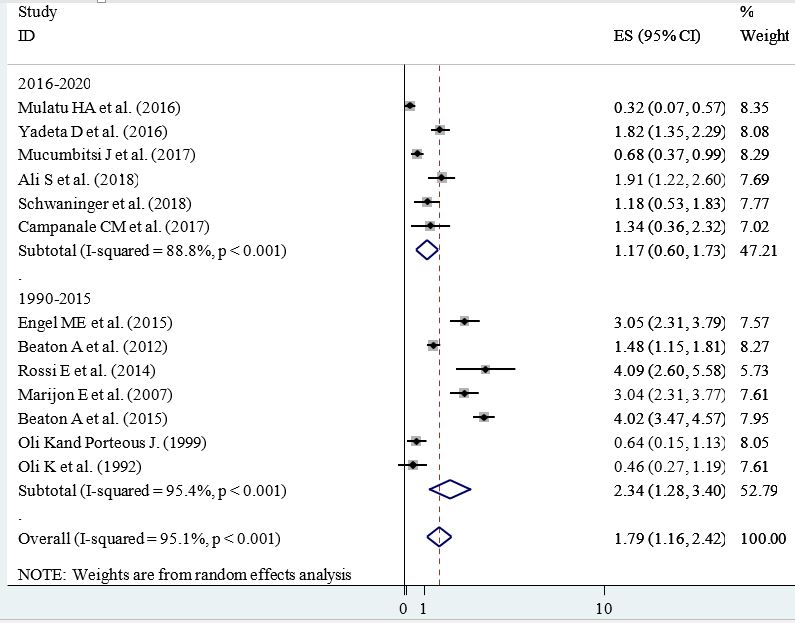
forest plot of subgroup analysis by year of publication showing the prevalence of rheumatic heart disease per 100 school children from 1990-2015 and 2016-2020 in East Africa, 2020

**Figure 5:**
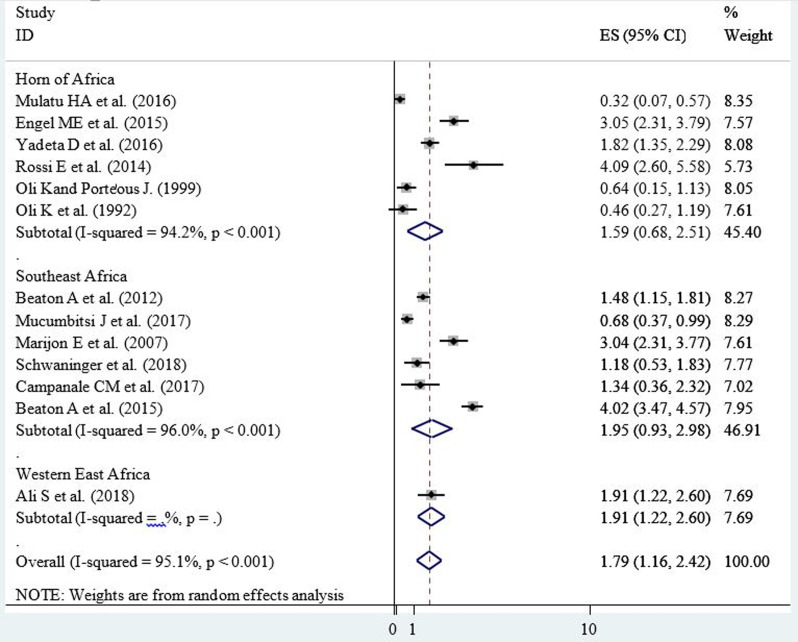
forest plot of subgroup analysis by study area category showing the prevalence of rheumatic heart disease per 100 school children in the horn, Western and Southeast parts of East Africa, 2020

**Sub-group analysis:** sub-group analysis was conducted by study area category and year of publication. Regarding the study area, countries of East Africa were grouped in three categories as the horn of Africa (Ethiopia, Eritrea, Djibouti and Somalia), Western East Africa (South Sudan) and Southeast Africa; containing the remaining east African countries. Accordingly, the horn of Africa had the lowest prevalence of RHD in school children among east African countries (Figure 4). Additionally, included articles were grouped into two categories (from 1990-2015 and 2016-2020) based on their year of publication. Thus, the lower prevalence of RHD in school children (11.7 cases per 1000 children, 95% CI=6.0, 17.3, I^2^=88.8%, p<0.001) was reported among studies published from 2016 to 2020 (Figure 5).

## Discussion

This study was aimed to estimate the prevalence of rheumatic heart disease (RHD) among school children in East Africa. Studies addressing the issue of RHD among school children in East Africa were limited. Hence, a total of thirteen studies were included in the final analysis. The included studies had used both clinical screening and echocardiographic confirmation of RHD in school children by using either WHO or World Heart Federation (WHF) criteria for defining RHD cases. The pooled result from those thirteen studies revealed that the prevalence of RHD in East Africa to be 1.79% (17.9 cases per 1000 school children). Comparable results were reported from other studies conducted in Nigeria [33] and Senegal [34]. This comparability might be due to similarities in socio-economic status between those African countries. Studies conducted in Pakistan [35], India [36] and New Zealand [37] had also reported nearly similar findings. A global systematic review and meta-analysis of population-based studies conducted by Noubiap JJ *et al*. had revealed that the pooled prevalence (from studies conducted in children and adults) of RHD ranging from 5.2% to 26.1% depending on the diagnostic criteria and procedure used; with a higher prevalence of RHD observed in Africa; which was nearly similar with findings of our meta-analysis [38].

The results of this systematic review and meta-analysis were lower than studies conducted in Yemen [39] and Tonga [40], which might be due to differences in study design. Studies in Yemen and Tonga were single observational studies, which may not indicate an overall prevalence of RHD. On the other hand, findings from this meta-analysis were higher than the estimated prevalence of RHD in developed countries like the United States (0.1-0.4 cases per 1000 children) [10]. This might be due to differences in socioeconomic status. RHD is a poverty-related non-communicable disease expected to be highly prevalent in developing countries like East Africa [5,11,12]. Developing countries lack appropriate case detection systems and treatment strategies, possibly due to limited resources. Community awareness and adherence to RHD preventive strategies are also expected to be low in developing countries; including countries of East Africa [14]. Those factors in combination might be associated with the observed higher prevalence of RHD in East Africa.

The sub-group analysis conducted by the study area category had revealed countries in the horn of Africa to have the lowest prevalence of RHD among school children than the rest of East African countries. This might be due to differences in diagnostic criteria they used; as only two of the six studies in the horn of Africa had used the WHF criteria, while five of the seven studies in East African countries had used the WHF criteria for diagnosing RHD. Literature had outlined that the WHF criteria are more sensitive than the WHO criteria for diagnosing RHD and results in a higher prevalence of RHD [38]. Another possible explanation might be due to differences in diagnosing procedure; as all the six studies in the horn of Africa had used auscultation for screening RHD cases and then confirm by echocardiography, while five of the seven studies in other East African countries used echocardiography both for screening and confirmation of RHD cases. Literature had shown that using echocardiography for screening and confirmation results in a higher prevalence of RHD than using auscultation for screening and echocardiography for confirmation [38,41]. Additionally, sub-group analysis by year of publication had shown a lower prevalence of RHD to be recorded among studies published after 2015. This might be due to increased health care services and technological advancements. Another possible explanation might be the increased interventions carried out to reduce the prevalence of RHD in Africa.

In 2015, the social cluster of the Africa Union commission has hosted a consultation with RHD experts in Addis Ababa, Ethiopia, to develop a roadmap for eliminating RHD from Africa. Accordingly, seven key actions were indicated by the Addis Ababa communique: 1) measuring the prevalence of RHD through prospective disease registry systems; 2) ensuring primary and secondary prevention of RHD; 3) improving access to reproductive health services for women with RHD; 4) technical expert and technological decentralization for diagnosing and treating RHD or acute rheumatic fever; 5) establishing national and regional centers for treatment and training; 6) initiating multi-sectoral RHD programs; and 7) resource mobilization through partnerships with international organizations [42]. So, the decreased prevalence of RHD after 2015 might be associated with the implementation of those key actions. But, the Addis Ababa communique had missed community mobilization, which is an essential principle to decrease the prevalence of RHD. Authors of this meta-analysis believe that communities´ perception, knowledge of RHD severity and strep throat complication and increased health-seeking behavior for strep throat are crucial points to eradicate/reduce RHD [43]. Despite this, acute respiratory illnesses including tonsillopharyngitis (strep throat) are considered mild and self-limiting diseases and health-seeking behavior for those illnesses is low in developing countries including in East Africa [13,14].

Despite a drastic drop in the prevalence of rheumatic heart disease in high-income countries, the observed prevalence of rheumatic heart disease in East Africa was high and it is known that RHD is a preventable cause for heart failure. A systematic review conducted in sub-Saharan Africa showed that about 14% of heart failure in the area is attributable to RHD [44]. Hence, preventing the occurrence and progression of RHD through the improvement of socio-economic and environmental conditions of at-risk populations (primordial prevention), early identification and treatment of strep throat (primary prevention), prevention of recurrent strep throat or reduction of progression by using antibiotic prophylaxis (secondary prevention) and medical and/or surgical treatment of advanced RHD cases (tertiary prevention) might be crucial to reduce the effect of this disease [12,45]. But authors of this meta-analysis believe that for low-income countries like East Africa, early identification and treatment of strep throat, early screening of silent rheumatic fever and/or RHD cases and provision of antibiotic prophylaxis are more important and feasible strategies than medical and/or surgical treatment of advanced RHD cases. Therefore, those feasible strategies should be strongly implemented to decrease the observed higher prevalence of RHD in East Africa. Furthermore, community mobilization and education to increase knowledge on strep throat and its complications and the effects of RHD are crucial to increase health-seeking behavior for strep throat, which in turn might be essential to reduce the observed higher prevalence of RHD in East Africa.

The roadmap for eliminating RHD from Africa developed in 2015 [41] should also consider and incorporate community mobilization and mass-media education on strep throat and its complications as a key action for reducing the higher prevalence of RHD in East Africa. Though the methodological techniques used in this meta-analysis were robust, its findings should be interpreted with considering the following concerns: 1) substantial heterogeneity on the prevalence of RHD was observed among the included studies; 2) this study includes echocardiographic studies which might overestimate the actual burden of RHD; 3) this study relies on a small number of studies with small sample size; 4) this study might not be free from bias as all works of literature might not be included. Nevertheless, this meta-analysis has important strengths; as authors had used robust meta-analytic techniques and internationally qualified tools for appraising the quality of included studies. Authors had also employed trim-fill analysis to minimize the impact of publication bias.

## Conclusion

The prevalence of rheumatic heart disease (RHD) among school children was found to be 1.79% (nearly 18 cases per 1000 children) in East Africa, which was considerably higher than results from developed countries. Therefore, experiences from developed countries should be adapted and implemented in East Africa. Community education on strep throat and its complication should be implemented through mass-media; even clear case definitions for strep throat should be developed and disseminated for the community to increase health-seeking behavior and to strengthen primary prevention of RHD. Rheumatic heart disease preventive strategies should be integrated with schools by training school teachers for early detection of strep throat and rheumatic fever. Further researches addressing factors associated with the high prevalence of RHD should also be encouraged.

### What is known about this topic

- Worldwide, more than 30 million people are thought to be victims of rheumatic heart disease (RHD);
- It is also a cause for an estimated 305,000 deaths and 11.5 million disabilities;
- About 84% of RHD cases and 80% of estimated deaths are contributed from Africa, South-East Asia and Western Pacific regions; RHD can occur at any ages, but school children (children aged from 5-15 years) are the highly affected groups.

### What this study adds

- The prevalence of rheumatic heart disease (RHD) among school children was found to be 1.79% (nearly 18 cases per 1000 children) in East Africa, which was considerably higher than results from high income countries;
- The horn of Africa had the lowest prevalence of RHD in school children among East African countries;
- To decrease the prevalence of rheumatic heart disease (RHD) in school children, it is better if RHD preventive strategies are integrated with schools by training school teachers for early detection of strep throat and rheumatic fever.
